# Assessment of quality of life in Gestational diabetes mellitus care – results of the pre-test of the disease-specific questionnaire GDM-QOL

**DOI:** 10.1186/s41687-026-01031-2

**Published:** 2026-03-14

**Authors:** Lisa Güldner, Klara Greffin, Holger Muehlan, Johannes Stubert

**Affiliations:** 1 University Gynecological Hospital and Polyclinic, University Medicine Rostock, 18059 Rostock, Germany; 2 Department Health and Prevention, Institute of Psychology, University of Greifswald, 17489 Greifswald, Germany; 3 German Center for Child and Adolescent Health, Greifswald/Rostock, Germany; 4 Department for Medical Psychology, Institute for Human Medicine, Health & Medical University Erfurt, 99084 Erfurt, Germany

**Keywords:** Quality of life, Gestational diabetes mellitus, Pregnancy, Maternal well-being, Hyperglycemia, Instrument development, Patient-reported outcome

## Abstract

**Introduction:**

Gestational diabetes mellitus (GDM) is the most common medical complication in pregnancy. Its onset puts a heavy strain on the positive connotation of pregnancy and is accompanied by feelings of fear, uncertainty, stress, a sense of losing control, a poorer perception of health and a reduced quality of life (QoL). QoL has a significant impact on the success of therapy because of its link to adherence and clinical outcomes. Therefore, its measurement has been suggested to be implemented as a clinical standard in GDM care. Since the Persian GDMQ-36 is the only GDM-specific questionnaire so far, the aim of our study was to develop a disease-specific QoL questionnaire in German.

**Methods:**

After translating the GDMQ36, we assessed the comprehensibility as well as face and content validity by an expert rating (*n* = 20) and conducted two pre-tests, including cognitive debriefings and structured interviews with women suffering from GDM (*n* = 30, *n* = 12).

**Results:**

The translated questionnaire had to be adapted and revised due to linguistic and cultural comprehension difficulties. Based on the findings of our qualitative studies, 33 questions about missing aspects were added, while most existing phrases needed to be modified or some even excluded. The preliminary version of the questionnaire contains 69 items within three domains (physical, psychological, social) and is subdivided into six facets (medical, treatment associated, emotional, behavioral, motivational, and social aspects).

**Conclusion:**

The questionnaire has the potential to provide a more comprehensive overview of women’s expectations, needs, impairments, and problems related to illness and treatment. It is planned, to prospectively test the newly developed GDM-QOL questionnaire to evaluate its psychometric performance in terms of validity and reliability.

**Supplementary Information:**

The online version contains supplementary material available at 10.1186/s41687-026-01031-2

## Background

Gestational diabetes is the most common medical complication in pregnancy and its prevalence is continuously increasing in the context of the global epidemic of obesity and diabetes. According to the International Diabetes Federation, 16.7% of pregnancies were affected by hyperglycemia in 2021, of which 80.3% were due to GDM [1]. In Germany, the prevalence of GDM added up to 13.2% in 2014/15 [2]. These results align with our own observations at our tertiary care center, where 14.9% of pregnant women were affected by GDM in 2017. In 2020, this percentage had increased to 18.9% and in 2021 to 23.6% (unpublished data).

The onset of GDM puts a heavy strain on the positive connotation of pregnancy for any woman and is accompanied by feelings of fear, anxiety, uncertainty, a sense of losing control, elevated levels of stress, a poorer perception of one’s health, reduced self-worth, and an impaired quality of life (QoL) [3–5]. Pregnant women perceive GDM as a powerful stressor based on the disease’s adverse impacts on all areas of their lives, and the threat it poses on them and their children by means of potential complications and long-term consequences [6]. To minimize these risks, consistent metabolic control is mandatory, which requires the women’s willingness to take an active part in treatment. At this juncture, it is inevitable to acknowledge the women’s personal viewpoint on their condition, and to consider any impairments and problems related to their disease and its treatment, since GDM adversely influences their lifestyle as well as their psychological comfort and well-being [7, 8]. Especially in lifestyle diseases such as diabetes and GDM, which require a high degree of self-management, a patient’s QoL has a significant impact on the success of therapy due to its link to adherence and clinical outcomes [7, 9].

While QoL is a meaningful health indicator, a disease-specific QoL (DisQoL) questionnaire tailored to women with GDM does not exist in German-speaking countries. In the past years, research on QoL in GDM has gained momentum, whereupon primarily generic and health-related but also diabetes-specific QoL questionnaires have been used for its assessment [10]. This comes at the cost that important aspects of health-related QoL (HrQoL) within GDM are not sufficiently represented. Accordingly, it appears necessary to provide a solid basis for further research by developing and validating an appropriate patient-reported outcome measure (PROM) for QoL-assessments in GDM. Therefore, it was the aim of our study to develop a GDM-specific QoL questionnaire [11]. To ensure its relevance and feasibility for the target group, we conducted a series of pre-tests of the newly developed GDM-QOL questionnaire.

## Methods

### The Persian GDMQ-36

To our knowledge, the GDMQ-36 by Mokhlesi et al. is the only questionnaire so far for assessing the disease-specific QoL in women with GDM [12]. It contains 36 items and consists of five domains: (1) concerns about high-risk pregnancy, (2) perceived constraints, (3) complications of GDM, (4) medication and treatment, as well as (5) support. Answers are given based on a 5-point-Likert-scale ranging from 1 (“strongly agree”) to 5 (“strongly disagree”), whereby a higher domain score indicates a higher HrQoL. As the Persian GDMQ-36 has been proven to be reliable and valid for the Iranian population, we aimed to test if the German version of the questionnaire would perform similarly after a precise translation and cultural adaptation, enabling its use in Germany.

This paper covers step (1) to (3) of the multi-staged development of our GDM-specific QoL-questionnaire:

1. Translation of the Persian questionnaire GDMQ-36 into German
2. Pre-testing with cognitive debriefings with women suffering from GDM and an expert rating for the evaluation of comprehensibility, face and content validity
3. Independent peer-consolidation process
4. Pilot testing of the preliminary questionnaire version
5. Validation of the questionnaire to investigate the psychometric performance of the measure in terms of reliability and validity

### Translation process

After gaining the permission from the Teheran-based research team, we got sent the original Persian QoL questionnaire GDMQ-36 as well as the corresponding scoring procedure, which were not included in the original paper [12]. To keep the deviations from the original as minor as possible, the emphasis has been put on the literal translation of the questions. Good comprehensibility and meaningfulness had to be ensured, while the initial content of every question had to be reflected preferably exact. The following three steps were performed:

***Step I****: Forward translation from Persian into German and synthesis of the German translations:* Firstly, the Persian GDMQ-36 was systematically forward translated from Persian into German by two Persian native speakers following the translation guidelines of the European Social Survey (2022) which suggests an independent and simultaneous translation by different persons [13]. Hereafter, the individual translations have been compared and differences have been discussed by the instrument developers as a team. Within this review process, they finally reached an agreement on a preliminary German version of the questionnaire.

***Step II****: Backward translation from German version into Persian:* Secondly, a third Persian native speaker and a registered translator for Persian, certified after ISO 17100, independently translated the preliminary German version of the GDMQ-36 back into Persian.

***Step III****: Review of the backward translated and original Persian version by a registered translator and development of a pre-final German version:* Subsequently, the registered translator compared the two backward translated Persian versions with the original Persian GDMQ-36 to determine any differences between them aiming to eliminate inconsistencies and inaccuracies in the preliminary German version. Conclusively, this German version of the GDMQ-36 underwent further small modifications with regard to content-related plausibility.

### Study participants

Study participants have been recruited by the attending obstetricians at our outpatient care facility at the Department of Obstetrics and Gynecology of the Rostock University Medical Center. All participants had previously been diagnosed with a GDM using a 75 g oral glucose tolerance test (oGTT) according to the International Association of the Diabetes and Pregnancy Study Groups (IADPSG) criteria [14]. Pregnant women were invited to participate in the study if they met the following inclusion criteria: they must have had a diagnosis of GDM within the current pregnancy, been at least 18 years old, had sufficient knowledge of the German language, and must not have any cognitive impairments. Women with a history of GDM being diagnosed once more in the current pregnancy have likewise been included, while their prior history regarding GDM was registered. Exclusion criteria included preexisting type I or type II diabetes mellitus as well as a known insulin resistance prior to pregnancy.

To represent the general GDM population as realistic as possible and to ensure an increased generalizability of our results, we have interviewed GDM-patients coming from various sociodemographic backgrounds and have taken into account the common distribution of the following main factors in our outpatient care facility, which is 58.0% vs. 42.0% in each case:

- normal weight (body mass index 18.5 to 24.9 kg/m^2^) vs. overweight/obesity (body mass index ≥ 25 kg/m^2^ before pregnancy)
- multiparity vs. nulliparity
- dietary vs. insulin treatment

The study respected the ethical standards of the Declaration of Helsinki and was given the approval of the local Ethics Committee (registration number A 2021–0142). After comprehensive information about the aims and procedure of the study, the women gave their written consent to the participation in the cognitive debriefing and to the assessment of their socio-demographic and medical data. All obtained data were housed in a secure manner in the university hospital.

### Pre-testing of the preliminary gGDMQ-36

For the pre-testing of the gGDMQ-36, we chose a qualitative approach. Firstly, we performed the cognitive debriefing method “think aloud” and structured interviews with women suffering from GDM. Subsequently, an expert survey with medical professionals was conducted. This was followed by a second round of cognitive debriefings with women suffering from GDM. These qualitative steps were used to adopt the initial conceptual model, which formed the basis for our measurement model and provided the content of the wording of items.

#### Cognitive debriefings and structured interviews

I. In order to examine the comprehensibility and linguistic appropriateness, the gGDMQ-36 was given to a group of 30 women with GDM within the setting of a cognitive debriefing for the first time [15]. In the course of a structured interview that was performed by a doctoral candidate, the women were inquired to read each question out loud, reply to it and justify their answer in order to investigate a profound understanding of each item. Subsequently, the women got asked to evaluate the comprehensibility of the items, the appropriateness of the language for laypersons and whether they had any difficulties regarding the questionnaire’s instructions. Additionally, they stated whether all relevant topics influencing their GDM-specific QoL were covered by the preliminary gGDMQ-36 and had the opportunity to mention missing aspects. Moreover, the women’s socio-demographic and medical data were collected via two master data sheets.
II. After a cultural and linguistic adaptation of the questionnaire, we performed a second round of cognitive debriefings (at least *n* = 10 patients) using the same approach as described above to investigate the comprehensibility and linguistic appropriateness of the revised gGDMQ-36. Women suffering from GDM, who had been included in the first round of cognitive debriefings, did not take part in the second round.

The evaluation followed a standardized process: initially, the clarity of the instructions and the possible answer options of the questionnaire were assessed. By analyzing the transcripts of the structured interviews, difficulties in understanding were identified. This included reading difficulties, like stuttering or repetition of syllables in certain words, hesitation in the reading flow before certain words, incorrect pronunciation or mispronunciation (e. g., emphasis on the wrong syllables), and spontaneous questions about the meaning of certain words or sentences. Problematic items were recorded, along with suggestions for improvements and desired thematic additions or deletions.

#### Expert rating

For the examination of content validity, an expert rating with medical specialists comprising gynecologists/obstetricians, endocrinologists/diabetologists, nutritionists, dieticians, midwives, nurses of the maternity ward and those specialized on diabetes, each with perennially years of work experience, was conducted. In total, we aimed for *n* = 20 experts, of which approximately half should have been practicing inside our own obstetric or endocrinology department and half in various extern obstetric clinics in cities throughout Germany [15].

Being invited to participate in our expert rating by the chief senior physician of the gynecological-obstetrical department, they were asked to assess the content relevance of each question of the preliminary gGDMQ-36 by means of a four-point-Likert-scale ranging from 1 (“not relevant at all”) to 4 (“very relevant”). By dividing the number of questions rated with 3 or 4 points by the total number of participating experts, the content validity index (CVI) according to Waltz and Baussell (1981) was calculated [16]. A question was considered sufficiently valid in terms of content and relevance if its CVI was > 0.79. Additionally, the scale-level content validity index (S-CVI) as the average of the individual question's CVI was calculated, whereby an S-CVI > 0.9 was considered acceptable [16]. Conclusively, suggestions for improvement and thematic additions to the questionnaire were obtained by medical specialists.

#### Readability assessment

The readability was assessed applying the Flesch-Reading-Ease-Index (FRE), a numeric value ranging between 0 and 100 in English texts [17]. The higher the value, the easier the text is to understand. Fundamentally, it is assumed that short sentences and words are noticeably easier to understand for the reader. By means of the calculated scoring, it can be ascertained whether a text will be appropriate and comprehensible for a specific target group [17].

For its use with German texts, a word factor had to be implemented into the Index’ original formula due to longer words in the German language [18]. Besides that, the ASW (Average Number of Syllables per Word) and ASL (Average Sentence Length) are pivotal values for the FRE’s calculation:

- *ASW*: total count of syllables in the text divided by its total word count
- *ASL*: total word count of a text divided by the total number of its sentences [17]

$$FRE German = 180 - ASL - \left({58.5 \times ASW} \right)$$

To determine the readability of our preliminary questionnaire, the FRE was calculated at two time points:

I. immediately after the translation of the Persian GDMQ-36 into German without undergoing any further editing and
II. after it has passed the process of pre-testing and independent peer-consolidation.

### Peer-consolidation and harmonization process

Based on the findings from the qualitative studies, the pre-test, and the expert survey, the resulting item pool was subsequently harmonized in terms of language, content, and representation of the concept by the members of the research group. Linguistic harmonization ensures that the items are consistently formulated in terms of language (e.g., using similar level of complexity). Content harmonization ensures that the item content is neither redundant nor ambiguous and that it does not combine multiple distinct meanings. Conceptual harmonization ensures that the item content comprehensively covers all identified domains and that they can be clearly assigned to the respective conceptual categories.

## Results

### Translation of the Persian questionnaire GDMQ-36

The process of forward and backward translation is depicted in Supplementary Figure 1. During the comparison of the two forward translations within the review process, the instrument development team easily agreed on a combined German version of the GDMQ-36 since they found no major differences in the two forward translations from Persian into German. The same was the matter when the two backward translations by the third Persian native speaker and the registered translator were compared to the Persian original questionnaire to eliminate inconsistencies and inaccuracies in the translation of the preliminary German version. This second review resulted in small modifications as the exchange of single words. The only phrases being entirely reformulated by the registered translator were “My need for sexuality has decreased.” into “I have less sexual intercourse.” (no. 13), “To measure my fasting blood sugar level, I have to stick to greater intervals between meals.” into “[…], I have to fast long and endure hunger.” (no. 19), “I have increased urination.” into “I often have to go to the toilet.” (no. 21) and “My blood sugar level has decreased” into “I have experienced hypoglycemia.” (no. 25). While the initial content of every question has still been reflected as exactly as possible, the rewording optimized the items’ comprehensibility as the phrases were less medically expressed and not as bulky in German language after the revisions made by the Persian translator. Therefore, the instrument developer team decided in a consultation to maintain the changes.

### Study participants

Medical and socio-demographic characteristics of participants are presented in Tables 1 and 2.

**Table 1:** Medical characteristics of study participants

Characteristic	Cognitive Debriefing I (n = 30)	Cognitive Debriefing II (n = 12)
Age, years Mean ± *SD* Min – Max	32 ± 5 23–41	33 ± 5 21–40
Pregravid body mass index, kg/m^2^ Mean ± *SD* Min - Max	31 ± 7 20–43	30 ± 8 20–50
Gestational age at GDM diagnosis, days Mean ± *SD* Min - Max	142 ± 49 51–208	168 ± 26 105–203
Gestational age at pretest, days Mean ± *SD* Min - Max	219 ± 38 126–266	235 ± 25 201–272
Gravidity, *n* Median Min – Max	2 1–6	2 1–6
Parity, *n* Median Min – Max	1 0–3	1 0–3
Nulliparity, *n* (%)	14 (46.7%)	6 (50.0%)
Planned pregnancy, *n* (%)	26 (86.7%)	9 (75.0%)
Insulin treatment, *n* (%)	18 (60.0%)	9 (75.0%)
Previous GDM, *n* (%)	6 (20.0%)	2 (16.7%)
Family history for diabetes mellitus type II, *n* (%)	17 (56.7%)	7 (58.3%)
Chronic Hypertension, *n* (%)	5 (16.7%)	2 (16.7%)
Mental illness, *n* (%)	4 (13.3%)	0 (0.0%)

**Table 2:** Socio-demographic characteristics of study participants

Characteristic	Cognitive Debriefing I (n = 30)	Cognitive Debriefing II (n = 12)
Family status, *n* (%) single partnership married separated/divorced	0 (0.0%) 18 (60.0%) 12 (40.0%) 0 (0.0%)	1 (8.3%) 7 (58.4%) 3 (25.0%) 1 (8.3%)
Highest level of education, *n* (%) Secondary school High school College/University Doctoral candidate/postgraduate No response	16 (53.3%) 5 (16.7%) 8 (26.7%) 0 (0.0%) 1 (3.3%)	8 (66.7%) 0 (0.0%) 4 (33.3%) 0 (0.0%) 0 (0.0%)
Financial status, *n* (%) Poor/low-income workers Moderate/middle class Good/upper class	2 (6.7%) 25 (83.3%) 3 (10.0%)	2 (16.7%) 8 (66.7%) 2 (16.7%)
Employment status, *n* (%) Employed Maternity leave/housekeeper	10 (33.3%) 20 (66.7%)	6 (50.0%) 6 (50.0%)
German native speaker, *n* (%)	28 (93.3%)	11 (91.7%)

In total *n* = 30 women suffering from GDM were recruited for the first cognitive debriefing. The distribution of the three main factors obesity (50%), nulliparity (46.7%) and insulin treatment (40%) approximately met those of our gynecological outpatient department being about 42% each. The women were on average 32 years old, had a pregravid BMI of 31 ± 7 kg/m^2^ and were primipara being pregnant with their second child. 20% of the women have had GDM in a previous pregnancy and 56.7% of the women have a positive family history of diabetes mellitus type II. They were either in a partnership (60%) or married (40%), most reached secondary school as their highest level of education (53.3%), described their financial status as moderate (83.3%) and were on maternity leave (66.7%).

In the second cognitive debriefing with *n* = 12 women with GDM, the distribution of the two main factors obesity (50%) and nulliparity (50%) approximately met these of our gynecological outpatient department being about 42% each. Solely the parameter insulin treatment (25%) was not exactly met.

The women’s age was averaging at 33 years, the pregravid BMI was 30 ± 8 kg/m^2^ and they were also primipara being pregnant with their second child. 16.7% of the women have had GDM in a previous pregnancy and 58.3% of the women have a positive family history of diabetes mellitus type II. Most women were in a partnership (58.4%), reached secondary school as their highest level of education (66.7%), described their financial status as moderate (66.7%) and were employed or on maternity leave in equal parts (50%).

### Pre-testing of the preliminary gGDMQ-36

#### Cognitive debriefing I and structured interviews

Based on the findings of our qualitative studies, the majority of the Persian questionnaire’s phrases could not be transferred one to one after its translation into German (Supplementary Figure 2, Table 3). Except for eight items which could be easily adopted without any amendments, numerous questions needed to be modified and some even to be excluded. The two phrases ‘I’m concerned about fetal death.’ and ‘I’m concerned about loss of fetal movement.’ (nos. 4, 5) frightened and unsettled the pregnant women to such an extent, that we decided to exclude them. Beyond that, these two phrases showed the necessity for weakening the original wording of another question with a similar content (no. 6: ‘I’m concerned about fetal abnormalities.’). Based on further findings of paraphrasing and reading out loud, 22 questions were modified linguistically or culturally to increase readability and comprehensibility. The cultural adaptation applied for instance for the item ‘Prayer with God has helped me tolerate the disease.’ (no. 31) as German women ascribed a reduced value to religious believe than those from Iran. Hence, we shifted the questions focus to themes like distraction, periods of relaxation and conscious time-outs, which could be more suitable topics for Western communities. In items with medical terms, the structured interview revealed the women’s missing knowledge about their meaning, e. g., in ‘I have blood sugar drop.’ (no. 25) they were unaware about potential symptoms of hypoglycemia which would have been an essential requirement to evaluate its influence on their QoL. Therefore, a brief explanation of hypoglycemia symptoms was added as background information to this question. Due to their similar content, two individual questions got merged into one item (nos. 10, 11; nos. 34, 36). As the pregnant women had mentioned uncovered, relevant topics influencing their GDM-specific QoL, 21 questions about missing aspects were added. In total, our German questionnaire contained 53 items after the first cognitive debriefing.

**Table 3:** Changes of questionnaire after cognitive debriefing I

Item no.	Change	Item no.	Change
1	left as it was	19	adapted
2	adapted	20	adapted
3	left as it was	21	adapted
4	deleted	22	adapted
5	deleted	23	adapted
6	adapted	24	left as it was
7	left as it was	25	adapted
8	adapted	26	adapted
9	adapted	27	adapted
10	merged with no. 11	28	left as it was
11	merged with no. 10	29	adapted
12	adapted	30	adapted
13	adapted	31	adapted
14	adapted	32	adapted
15	adapted	33	left as it was
16	left as it was	34	merged with no. 36
17	adapted	35	left as it was
18	adapted	36	merged with no. 34

#### Expert rating

Overall, *n* = 20 medical professionals were recruited for the expert rating: around half of them (*n* = 9) from extern gynecological-obstetrical clinics in cities throughout Germany; the other half (*n* = 11) from our own gynecological-obstetrical and endocrinological department (Table 4).

**Table 4:** Medical professions participating in expert rating

Medical profession	Internal experts	External experts
Obstetrician, *n* (%)	4 (36.4%)	8 (88.9%)
Endocrinologist/Diabetologist, *n* (%)	0 (0.0%)	1 (11.1%)
Midwife, *n* (%)	1 (9.1%)	0 (0.0%)
Nurse of the maternity ward, *n* (%)	5 (45.5%)	0 (0.0%)
Nurse specialized on diabetes/dietician, *n* (%)	1 (9.1%)	0 (0.0%)
Total	11	9

Detailed results in terms of content validity are shown in Supplementary Figure 3 and Table 5. The S-CVI of the German GDMQ-36 amounted to 0.74. In total, 29 of 53 questions were rated as relevant with CVI > 0.79 and had therefore not to be changed. Nevertheless, four questions were slightly modified for an easier reading flow and two questions were merged because of their similar content. Of the other 24 questions rated with CVI < 0.79, eight required certain adjustments in wording, eleven were already excluded and five were left as they were. All of these five questions had a CVI > 0.65 and were of high relevance from the pregnant women’s point of view in the cognitive debriefing, which was why we kept them in the questionnaire in the first place.

**Table 5:** Changes of questionnaire after expert rating

Item no.	CVI	Change	Item no.	CVI	Change
1	1.00	-	28	0.35	deleted
2	0.95	adapted	29	0.45	deleted
3	0.70	adapted	30	0.85	-
4	0.75	deleted	31	0.90	-
5	0.95	adapted	32	0.90	adapted
6	0.40	deleted	33	0.55	-
7	0.90	-	34	0.65	deleted
8	0.70	adapted	35	0.90	-
9	0.30	adapted	36	0.90	adapted
10	0.80	-	37	0.40	deleted
11	0.85	-	38	0.65	adapted
12	0.95	-	39	0.85	-
13	0.70	adapted	40	0.95	-
14	0.80	-	41	0.80	-
15	0.75	left as it was	42	0.64	adapted
16	0.95	-	43	0.64	adapted
17	0.70	left as it was	44	0.32	deleted
18	0.75	left as it was	45	0.45	deleted
19	0.90	-	46	0.50	deleted
20	0.65	left as it was	47	0.55	deleted
21	0.80	-	48	0.70	left as it was
22	0.85	-	49	0.95	-
23	0.80	-	50	0.95	-
24	0.85	left as it was	51	0.95	left as it was
25	0.80	adapted	52	0.80	merged with no. 53
26	0.55	deleted	53	0.80	merged with no. 52
27	0.55	adapted			

S-CVI = 0.74

The eleven deleted items had a CVI between 0.30 and 0.55 in majority and concerned inter alia questions revolving around symptoms not exclusively relatable to GDM and more so to pregnancy itself or even a diabetes mellitus type I. Examples are ‘I repeatedly go to the bathroom.’, ‘I have feelings of thirst and dry mouth.’, ‘I get angry soon’ and ‘My sexual activity has decreased due to GDM.’ (nos. 26, 28, 29, 44). Two deleted items with CVI ≥ 0.75 were of low relevance for the pregnant women as hypoglycemia in their newborn was an unknown complication for most women and if they had to apply insulin, none of them ever had to adjust their insulin dose independently of medical recommendations (nos. 4, 34).

As for uncovered aspects of the women’s HrQoL, the medical specialists recommended adding questions revolving around topics like feelings of guilt against the fetus, feelings of being left alone and the pregnancy being not as lighthearted as before, but also positive aspects like the motivation to live a healthier life or the chance to influence the course of the disease positively through one’s behavior. This led to the addition of eight items (nos. 16, 25, 32, 40, 41, 42, 44, 48).

To sum up, 11 questions got deleted, two questions were merged into one and eight items were added in the process of the expert rating, leading to the preliminary German questionnaire comprising 49 items.

#### Cognitive debriefing II and structured interviews

Based on the findings of our second qualitative study, 28 of 49 items were left as they were, 15 required certain adjustments in wording, four were excluded and two were split up. In many cases, merely small modifications were required. Major adjustments were only necessary when abstractly formulated questions needed to be specified (nos. 38, 41, 42). In addition, phrases including words with a negative connotation were replaced through more positive ones, e. g, ‘Conversations and examinations during my regular prenatal appointments take away many worries’ was changed into ‘[…] calm me down and encourage me’ or ‘I feel left alone with this disease.’ was changed to ‘I would like to receive more support from my environment because I sometimes feel left alone with this disease.’ (nos. 36, 40). Further, four items had to be deleted as their statements made assumptions about certain circumstances, contained unknown complications of GDM or aroused fears that the women did not have before reading our questionnaire (nos. 5, 7, 38, 44). Since two items contained a duplicated question content, they were divided into two individual questions each: The first item dealt with difficulties associated with changing everyday habits regarding diet and exercise and the second one with problems in the technical handling of insulin syringes and blood glucose measurement (nos. 21, 30).

Missing aspects mentioned in the second cognitive debriefing covered topics such as the pregnant women’s self-management through exercise, the unborn child as a motivation for lifestyle adjustments, the feeling of sufficient information about their illness, the impairment caused by too much focus on nutrition and the additional time required for meal preparation. These aspects were included in the questionnaire by the addition of eight new questions (nos. 13, 14, 21, 25, 26, 28, 42, 43)(Supplementary Figure 4, Table 6).

**Table 6:** Changes of questionnaire after cognitive debriefing II

Item no.	Change	Item no.	Change
1	left as it was	26	left as it was
2	left as it was	27	left as it was
3	left as it was	28	left as it was
4	left as it was	29	left as it was
5	deleted	30	split: into nos. 45 & 52
6	left as it was	31	left as it was
7	deleted	32	left as it was
8	left as it was	33	left as it was
9	adapted	34	left as it was
10	left as it was	35	left as it was
11	left as it was	36	adapted
12	adapted	37	adapted
13	left as it was	38	deleted
14	left as it was	39	adapted
15	adapted	40	left as it was
16	left as it was	41	adapted
17	adapted	42	adapted
18	adapted	43	left as it was
19	left as it was	44	deleted
20	left as it was	45	left as it was
21	split: into nos. 22 & 23	46	left as it was
22	adapted	47	adapted
23	adapted	48	adapted
24	adapted	49	left as it was
25	left as it was		

#### Readability assessment

For computation of *Flesch-Reading-Ease-Index (FRE)*, the following parameters were assessed via “WordCreator” [19]:

I. total count of syllables: 1140 total count of words: 582 total number of sentences: 61

FRE _German_ = 180 - ASL - (58.5 * ASW) = 180–582/61 - (58.5 × 1,140/582) = 55.9

II. total count of syllables: 2398 total count of words: 1223 total number of sentences: 141

FRE = 180 - ASL - (58.5 * ASW) = 180–1223/141 - (58,5 × 2,398/1,223) = 74

Directly after the translation of the GDMQ-36 from Persian into German (I.), the FRE index showed a value of 56 which equals ‘medium-hard’. After completion of the independent peer-consolidation (II.), this value increased even higher and reached an FRE index of 74 equaling ‘medium-easy’. Having a value above 70, our German QoL-questionnaire would be appropriate also for readers having a lower educational level than pupils being 13–15 years old [17].

### Peer-consolidation and harmonization process

Due to the substantial changes to the original GDMQ-36 questionnaire in terms of the addition, deletion and modification of items, the original domains of the GDMQ-36 did not fully represent the item content. Hence, we had to restructure the preliminary version of the questionnaire within the independent peer-consolidation process. As a result, some new categories emerged, like motivation/activation as well as stigma/feelings of guilt. A separate domain which only covered aspects of insulin therapy was established. After doing so, our QoL questionnaire contained three domains (physical, psychological, social) and was subdivided into six facets (medical, treatment associated, emotional, behavioral, motivational, and social aspects) including four to ten items each.

As a next step, the questionnaire has been further revised including 15 adjustments in wording, one deletion and five split-ups (Supplementary Figure 5, Table 7). The deletion got necessary because item no. 36 covered support as an aspect that did not fit the general concept of the instrument. As five questions addressed two different concepts, they were split into two separate items (nos. 20, 31, 47 - 49). The newly added 11 questions predominantly cover aspects of physical activity: having not enough time or motivation to exercise, missing practical guidance or offers for physical activities in pregnancy, worries about pregnancy as a result of incorrect or too intense physical activity. One item was added each for the child’s later diabetes risk and the mother’s increased risk of cardiovascular diseases. The resulting version of our QoL questionnaire has undergone a number of significant changes. For this reason, we renamed it from GDMQ-36 to Gestational Diabetes Mellitus - Quality of Life (GDM-QOL) questionnaire.

**Table 7:** Changes of questionnaire after independent peer-consolidation

Item no.	Change	Item no.	Change
1	left as it was	29	left as it was
2	left as it was	30	adapted
3	left as it was	31	split: into nos. 13 & 14
4	left as it was	32	left as it was
5	left as it was	33	left as it was
6	left as it was	34	left as it was
7	adapted	35	adapted
8	adapted	36	deleted
9	adapted	37	left as it was
10	adapted	38	left as it was
11	left as it was	39	left as it was
12	adapted	40	left as it was
13	left as it was	41	left as it was
14	adapted	42	left as it was
15	adapted	43	left as it was
16	adapted	44	adapted
17	adapted	45	left as it was
18	left as it was	46	left as it was
19	adapted	47	split: into nos. 17 & 57
20	split: into nos. 55 & 56	48	split: into nos. 61 & 66
21	adapted	49	split: into nos. 59 & 65
22	adapted	50	left as it was
23	left as it was	51	left as it was
24	left as it was	52	left as it was
25	left as it was	53	left as it was
26	left as it was	54	left as it was
27	left as it was	55	left as it was
28	left as it was		

## Discussion

### Main findings

The main objective of this first part of our research project was the development of a German version of the GDMQ-36 questionnaire, which encompasses pregnancy- and disease-specific aspects of the QoL of women suffering from GDM. We accomplished a content-analytical validation through the evaluation of the performed pre-tests, a specification of the items’ formulation, the adaptation of items based on the results of the pre-tests being conducted on women suffering from GDM (face validity) and eventually the finalization of the questionnaire for the planned pilot study. The original 36-item measure, developed and validated in Iran [12], is, to our knowledge, the only validated questionnaire worldwide that specifically aligns with the interests of these women’s HrQoL and addresses its key dimensions. It has formed the basis for the development of a German-language version.

During the translation and pre-testing several challenges appeared: As the wording of certain items caused considerable difficulties in comprehension, rephrasing became necessary. This might be caused by the strong differences in language and culture. Moreover, some existing items were classified by experts as less relevant (item content validity index < 0.79). Finally, crucial aspects of HrQoL were identified that had not yet been covered by the original questionnaire. Taken together, these findings necessitated a fundamental revision of the questionnaire. Thus, a direct adaptation of the GDMQ-36 did not seem appropriate anymore. As a result, some of the original items were removed, new items were added, and several item wordings were refined. After carefully weighing the pros and cons of pursuing a highly equivalent adaptation, we decided to develop a largely new questionnaire, drawing on the initial item pool of the original GDMQ-36.

There were also some external reasons that backed up our decision: In the development of the original measure, 30 items were excluded based on an exploratory factor analysis using Varimax rotation. Thus, this selection step was guided primarily by statistical rather than conceptual or content-related criteria, and may therefore have failed to ensure that all relevant aspects were included in the final item pool. Given that the Persian GDMQ-36 was not translated or validated in any other language so far, it is not widely established as a standard reference instrument. Therefore, it seemed acceptable to forgo the missing comparability of the measures in favor of a conceptually saturated measurement approach.

In retrospect, one might ask whether developing the German instrument from scratch would have been a more appropriate approach than using an existing measure as a starting point. However, it is common practice in the development of PROMs to inform preliminary conceptual models through literature reviews and by examining existing PRO measures and items [20]. Moreover, some of the most widely established instruments are based on existing measures - for example, the SF-36 was derived from the MOS-100. Therefore, even if we had initially intended to develop a completely new measure, it is likely that content and items from the GDMQ-36 would have been included in the initial item pool. The same applies to the original Persian GDMQ-36, which incorporated nine items drawn from a literature review of various existing instruments (e.g., the Diabetes Management Self-Efficacy Scale).

Content-related, linguistic, and formal adjustments were made to optimize the content validity of the instrument for the German context. Following an independent peer-consolidation process—aimed at harmonizing the item pool in terms of language, complexity, and content—the preliminary German version of the GDM-QOL questionnaire comprised 69 items. These represent three core domains of health-related quality of life (physical, mental, and social), in accordance with the WHO’s multidimensional concept of health, and are further subdivided into six facets: medical, treatment-related, emotional, behavioral, motivational, and social aspects.

As a next step, the instrument requires independent pilot testing and validation to assess its psychometric properties. Following the successful validation, translation and cultural adaptation into other languages will be considered, paving the way for a broader use in research and clinical practice, and ultimately contributing to the improvement of GDM care.

### Limitations & strengths

Our study reveals some limitations: The pre-testing of this study was conducted as a single-center prospective study. Thus, the included study participants may not be completely representative of the population of women with GDM in Germany or even other German-speaking countries like Austria and Switzerland. It could also be conceivable that women refusing to participate in the study belong to a certain social, ethnic, or other vulnerable group that has been underrepresented in the findings of our study due to their avoidance of participation.

However, our study also has some strengths: Not only did we present a consistent forward-backward

translation of the original measure, but we also conducted several complementary studies. These allow for appropriate adaptation to the new cultural context. It was also possible to involve different stakeholders - patients, clinicians, researchers, as well as other expert groups. Finally, our study combines different methods, combining various qualitative and quantitative data collection and analysis approaches as well as using several linguistic and psychometric criteria for item evaluation.

### Conclusion

The development of our GDM-specific questionnaire marks an important step towards a more comprehensive understanding of the expectations, needs, and challenges faced by women with gestational diabetes mellitus (GDM). By focusing on health-related quality of life (HrQoL), this tool aims to support the provision of individualized treatment plans that incorporate tailored information, targeted support, and psychological counselling—elements that are essential for improving healthcare in lifestyle-related conditions such as GDM [7, 8].

The questionnaire has the potential to enable more sensitive comparisons between different established diagnostic and therapeutic approaches by evaluating their impact on HrQoL. It may help explain why treatments with similar clinical efficacy yield different outcomes in practice, as issues such as treatment burden or psychological distress can influence adherence. This could uncover previously overlooked factors leading to inconsistent therapy adherence or treatment discontinuation [7, 10].

Moreover, the instrument is designed to assess the effects of emerging innovations - such as continuous glucose monitoring, insulin pump therapy, and app-based monitoring - on pregnant women’s HrQoL. Multi-dimensional HrQoL assessment is vital for evaluating and optimizing new approaches in GDM care, ensuring that treatment strategies are aligned with patients’ real-world experiences and psychosocial needs [11].

## Electronic supplementary material

Below is the link to the electronic supplementary material.

Supplementary material 1

Supplementary material 2

## Data Availability

The datasets used and/or analyzed during the current study are available from the corresponding author upon reasonable request.

## Abbreviations

DisQoL: Disease-specific Quality of Life
FRE: Flesch-Reading-Ease-Index
GDM: Gestational Diabetes Mellitus
GDM-QOL: Gestational Diabetes Mellitus - Quality of Life
gGDMQ-36: [g]erman Gestational Diabetes Mellitus Questionnaire 36
HrQoL: Health-related Quality of Life
QoL: Quality of Life
