# Supplementary material for: Assessment of quality of life in Gestational diabetes mellitus care – results of the pre-test of the disease-specific questionnaire GDM-QOL
Source: J Patient Rep Outcomes. 2026 Mar 14;10:53. doi: 10.1186/s41687-026-01031-2 (PMC13065970; doi:10.1186/s41687-026-01031-2)
Supplement: Supplementary file 1 — Supplementary material 1 [file 41687_2026_1031_MOESM1_ESM.pptx]

## Slide 1
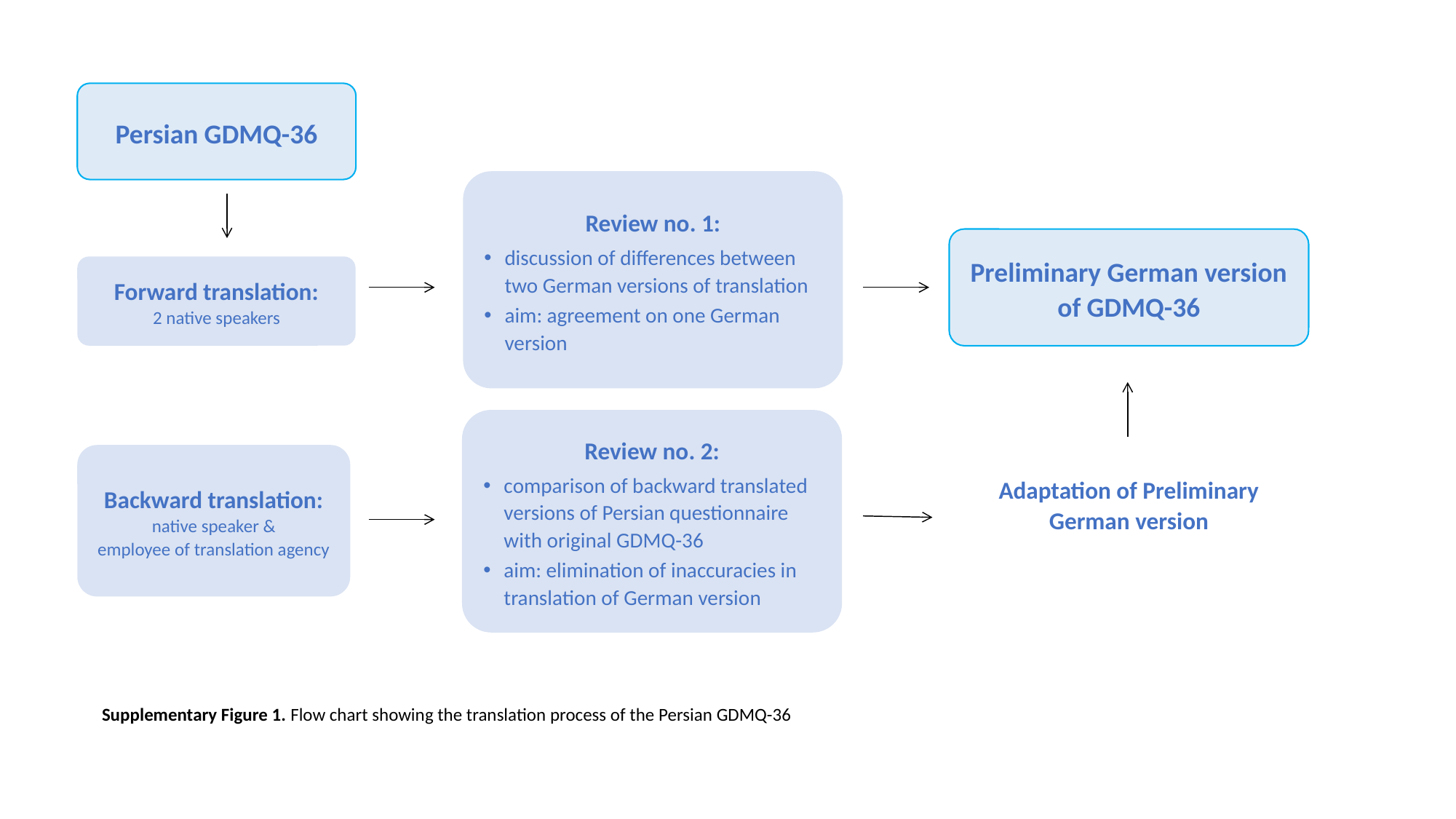

Persian GDMQ-36
Review no. 1:
discussion of differences between two German versions of translation
aim: agreement on one German version
Preliminary German version
of GDMQ-36
Forward translation:
2 native speakers
Review no. 2:
comparison of backward translated versions of Persian questionnaire with original GDMQ-36
aim: elimination of inaccuracies in translation of German version
Adaptation of Preliminary German version
Backward translation:
native speaker &
employee of translation agency
Supplementary Figure 1. Flow chart showing the translation process of the Persian GDMQ-36

## Slide 2
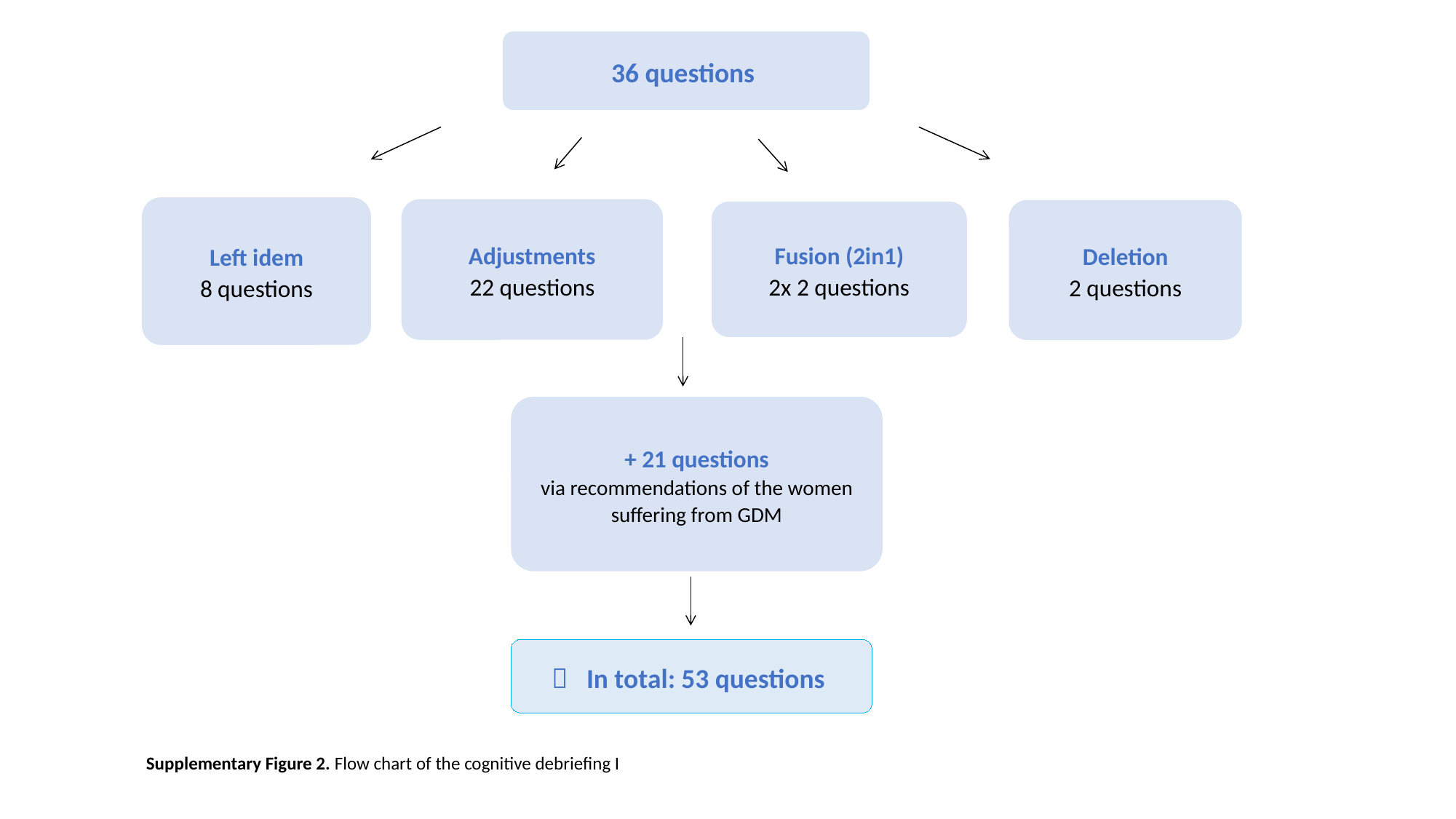

36 questions
Adjustments
22 questions
Fusion (2in1)
2x 2 questions
Deletion
2 questions
Left idem
8 questions
 In total: 53 questions
Supplementary Figure 2. Flow chart of the cognitive debriefing I
+ 21 questions
via recommendations of the women
suffering from GDM

## Slide 3
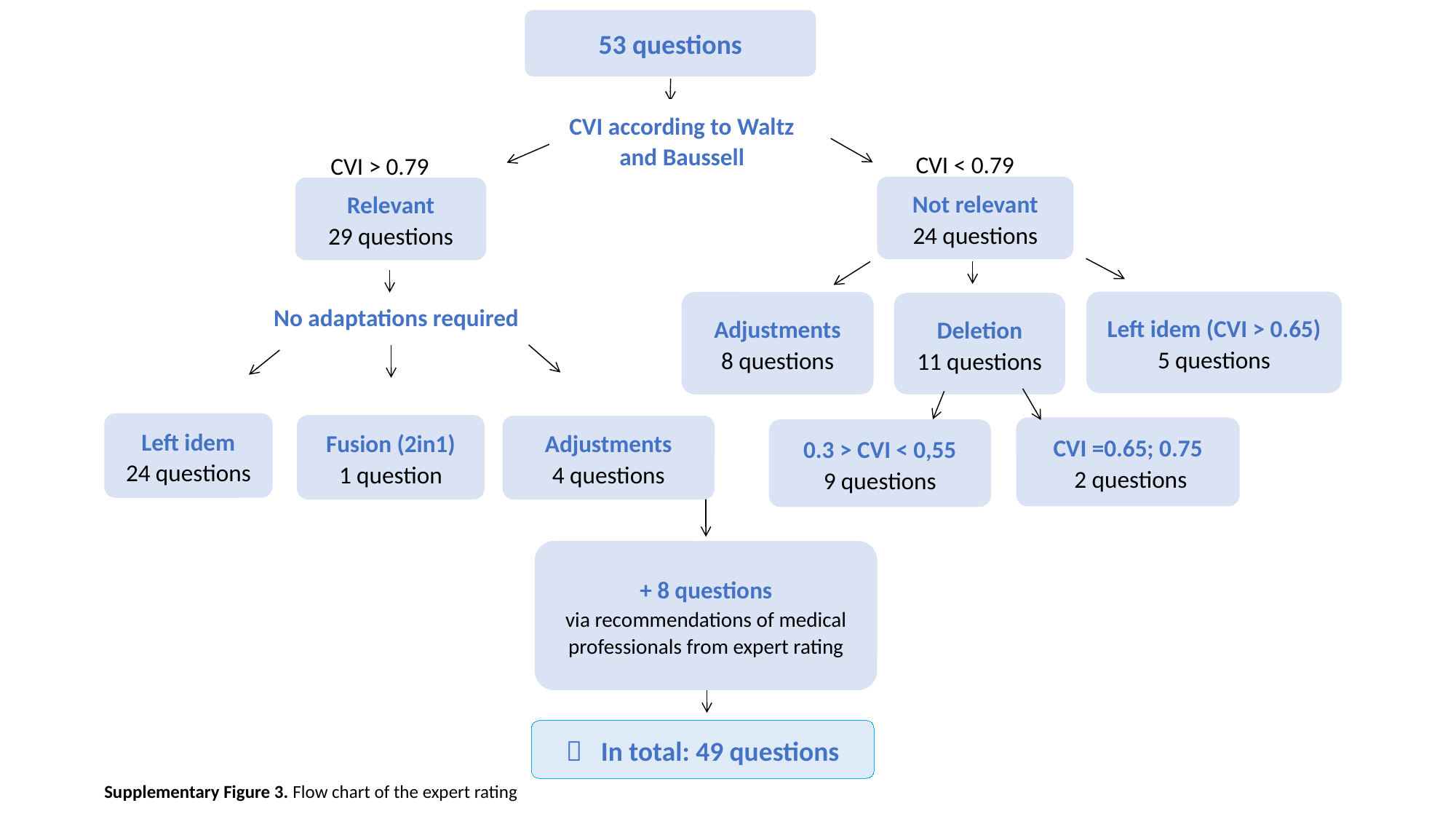

53 questions
CVI according to Waltz
and Baussell
CVI < 0.79
CVI > 0.79
Not relevant
24 questions
Relevant
29 questions
Left idem (CVI > 0.65)
5 questions
Adjustments
8 questions
Deletion
11 questions
No adaptations required
Adjustments
4 questions
CVI =0.65; 0.75
 2 questions
0.3 > CVI < 0,55
9 questions
Fusion (2in1)
1 question
Left idem
24 questions
+ 8 questions
via recommendations of medical professionals from expert rating
Supplementary Figure 3. Flow chart of the expert rating
 In total: 49 questions

## Slide 4
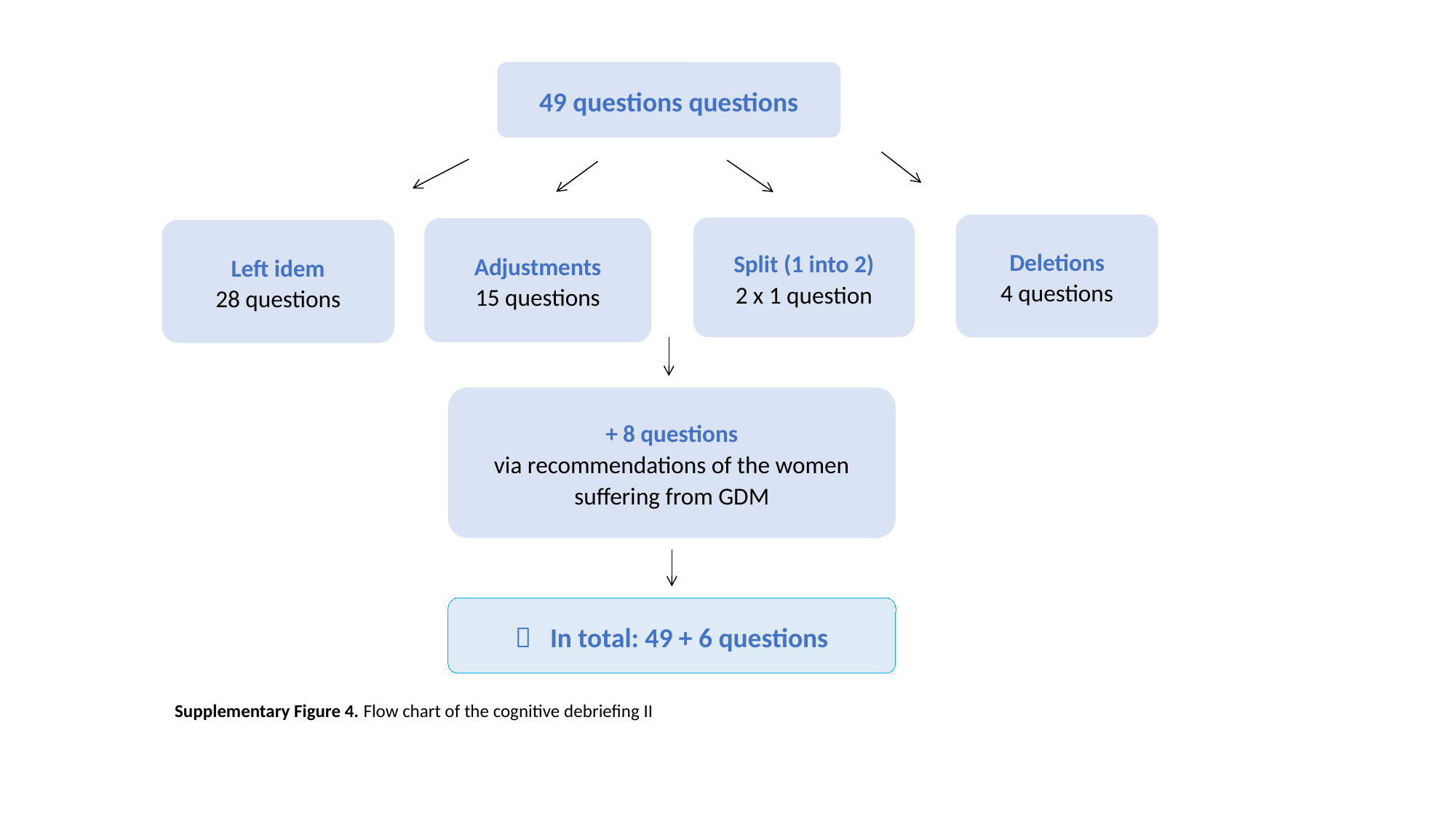

49 questions questions
Left idem
28 questions
Split (1 into 2)
2 x 1 question
Adjustments
15 questions
Deletions
4 questions
+ 8 questions
via recommendations of the women suffering from GDM
 In total: 49 + 6 questions
Supplementary Figure 4. Flow chart of the cognitive debriefing II

## Slide 5
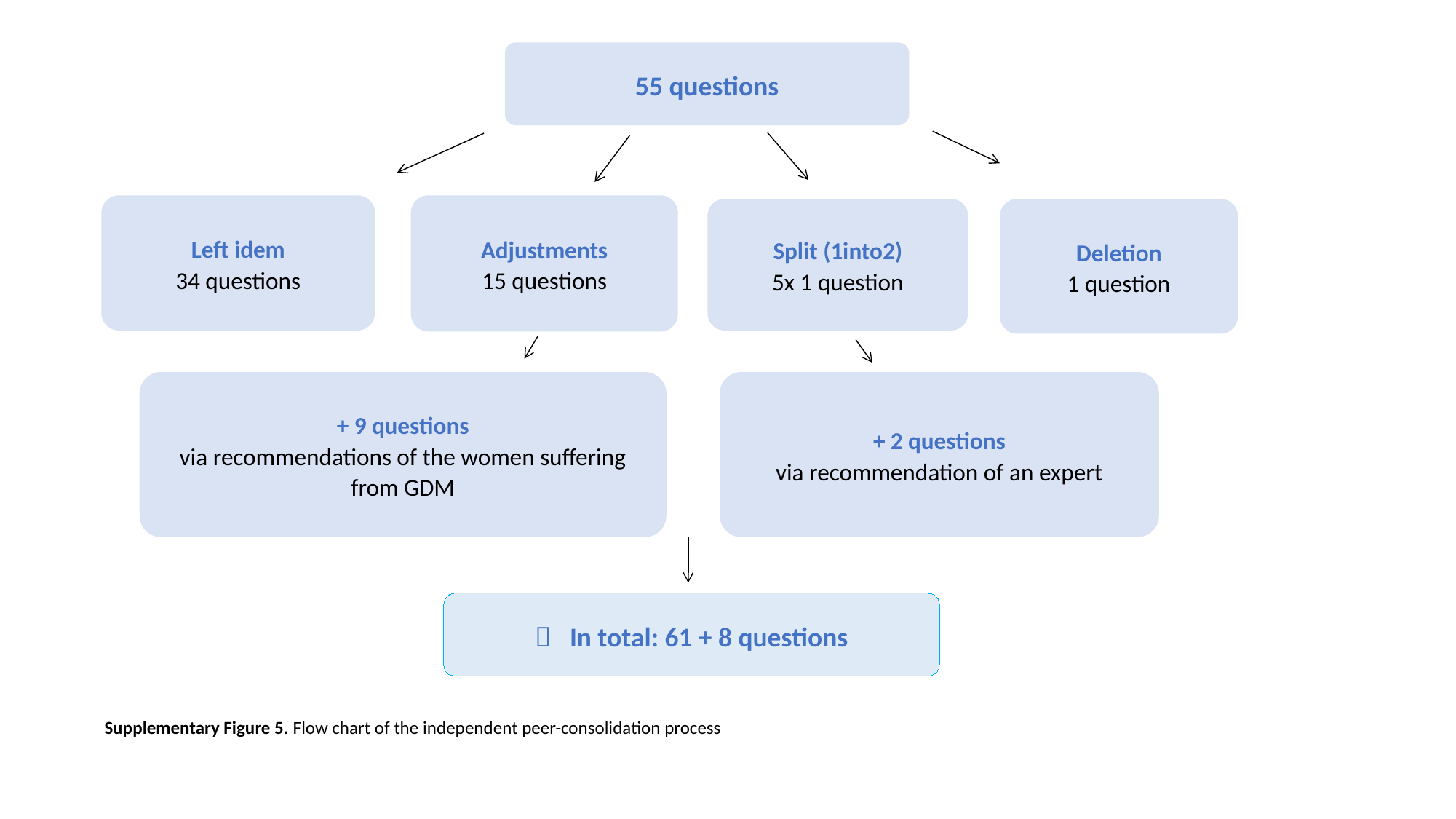

55 questions
Left idem
34 questions
Adjustments
15 questions
Split (1into2)
5x 1 question
Deletion
1 question
+ 9 questions
via recommendations of the women suffering from GDM
 In total: 61 + 8 questions
Supplementary Figure 5. Flow chart of the independent peer-consolidation process
+ 2 questions
via recommendation of an expert
