# Supplementary material for: Assessment of quality of life in Gestational diabetes mellitus care – results of the pre-test of the disease-specific questionnaire GDM-QOL
Source: J Patient Rep Outcomes. 2026 Mar 14;10:53. doi: 10.1186/s41687-026-01031-2 (PMC13065970; doi:10.1186/s41687-026-01031-2)
Supplement: Supplementary file 2 — Supplementary material 2 [file 41687_2026_1031_MOESM2_ESM.docx]

| **Supplementary Table 8.** Concepts captured in the preliminary Gestational Diabetes Mellitus - Quality of Life (GDM-QOL) questionnaire | | |
| --- | --- | --- |
|  | |  |
| **I. Physical Domain** | |  |
| **1.1 Medical Aspects** | |  |
| 1. **Unborn Child** | |  |
| *Aufgrund meines Schwangerschaftsdiabetes habe ich die Sorge, …* | | *worry about/ fear of* … |
| 1 | dass es meinem Kind durch diese Krankheit während der Schwangerschaft nicht gut geht. | fetal medical complications |
| 2 | dass sich das Gewicht meines ungeborenen Kindes zu stark erhöht. | macrosomia |
| 3 | vor einer Frühgeburt. | preterm delivery |
| 4 | dass mein Kind nach der Geburt von mir getrennt überwacht werden muss. | need for neonatal care unit |
| 5 | dass mein Kind später an Übergewicht leidet. | child being overweight |
| 6 | dass mein Kind später auch Diabetes bekommt. | child developing diabetes |
|  | |  |
| 1. **Mother** | |  |
| *Aufgrund meines Schwangerschaftsdiabetes habe ich die Sorge, …* | | *worry about/ fear of* … |
| 7 | dass die Geburt schwierig verläuft. | complicated delivery |
| 8 | dass ich in einer weiteren Schwangerschaft erneut einen Schwangerschaftsdiabetes entwickle. | GDM reoccurrence in subsequent pregnancies |
| 9 | dass ich in den nächsten Jahren dauerhaft an Diabetes erkranke. | developing diabetes mellitus type II |
| 10 | dass ich ein erhöhtes Risiko für einen Herzinfarkt und Schlaganfall habe. | suffering a heart attack or stroke |
|  | |  |
| **II. Psychological** | |  |
| **2.1 Emotional Aspects** | |  |
| 1. **Burdens & Constraints** | |  |
| *Aufgrund meines Schwangerschaftsdiabetes …* | | *diagnosis being accompanied by* *feelings of…* |
| 11 | ist meine Schwangerschaft weniger unbeschwert als vor der Diagnose. | fewer light-heartedness |
| 12 | ist meine Stimmung seit der Diagnose schlechter als zuvor. | lowered mood |
| 13 | fühle ich mich manchmal überfordert. | being overwhelmed |
| 14 | fühle ich mich manchmal hilflos. | helplessness |
| 15 | kreisen meine Gedanken zu viel um das Thema Ernährung. | circles of thought around food |
| 16 | nimmt diese Erkrankung zu viel Raum in meinem Denken ein. | thoughts being dominated by disease |
|  | |  |
| 1. **Stigmatization & Guilt** | |  |
| *Aufgrund meines Schwangerschaftsdiabetes…* | |  |
| 17 | habe ich bei hohen Blutzuckerwerten ein schlechtes Gewissen. | guilty conscience if blood sugar level is high |
| 18 | fühle ich mich dem Vorurteil ausgesetzt, dass ich mich vor meiner Schwangerschaft ungesund ernährt oder nicht ausreichend bewegt hätte. | stigmatization to have had an  unhealthy lifestyle before pregnancy |
| 19 | habe ich Schuldgefühle gegenüber meinem ungeborenen Kind. | feelings of guilt towards fetus |
| 20 | habe ich ein schlechtes Gewissen, wenn ich mich nicht genug bewege. | guilty conscience if not exercising enough |
|  | |  |
| **2.2 Behavioral Aspects** | |  |
| 1. **Exercise & Physical Activity** | |  |
| *Im Zusammenhang mit meinem Schwangerschaftsdiabetes…* | |  |
| 21 | fällt es mir schwer, mich mehr zu bewegen. | difficulties being more physically active |
| 22 | mangelt es mir an Zeit für Sport und körperliche Aktivität. | lack of time |
| 23 | mangelt es mir an Motivation und Willen für Sport und körperliche Aktivität. | lack of motivation |
| 24 | fehlt es mir an Anleitung, Sport und körperliche Aktivität praktisch umzusetzen. | lack of guidance |
| 25 | fehlt es mir an Angeboten für Sport und körperliche Aktivitäten in der Schwangerschaft. | lack of offers for exercise during pregnancy |
| 26 | habe ich die Sorge, durch falsche oder zu viel körperliche Aktivität die Schwangerschaft zu gefährden. | worries of jeopardizing the pregnancy trough wrong or too much physical activity |
| 27 | bin ich unsicher, welche körperlichen und sportlichen Aktivitäten in der Schwangerschaft gefahrlos möglich sind. | uncertainty about risk-free physical activities in pregnancy |
| 28 | fällt es mir schwer, mich mehr zu bewegen, da mein Umfeld mich hierbei nicht unterstützt. | lack of support trough environment as reason for minimal physical activity |
| 29 | belastet es mich, dass mein Umfeld sich nicht mit mir gemeinsam mehr bewegen möchte. | emotional burden of the surrounding not participating in movement |
|  | |  |
| 1. **Diet & Eating Behavior** | |  |
| *Im Zusammenhang mit meinem Schwangerschaftsdiabetes belastet es mich…* | | *burden of…* |
| 30 | Speisen und Obst nur in kleinen und festgelegten Portionen zu essen. | set portion sizes for meals and fruit |
| 31 | auf die Speisen und Früchte verzichten zu müssen, die ich früher gern gegessen habe. | sacrifice for favorite food |
| 32 | für mich selbst andere Mahlzeiten zuzubereiten als für meine Familie, da meine Ernährung von der meiner Familie abweicht. | preparing different meals for family and oneself |
| 33 | mehr Geld für den Einkauf der empfohlenen Lebensmittel ausgeben zu müssen. | higher costs for purchase of recommended diet |
| 34 | mehr Zeit für die Auswahl der Lebensmittel und die Planung meiner Mahlzeiten aufbringen zu müssen. | higher time investment for selection of food and for meal planning |
| 35 | entscheiden zu müssen, auf welche Lebensmittel ich lieber verzichten sollte. | decision of foods to avoid |
| 36 | selbst verzichten zu müssen, wenn andere Personen sich ungesund ernähren. | restraint in an unhealthy food consuming environment |
| 37 | dass mir Ideen und kreative Einfälle für eine gesunde und abwechslungsreiche Ernährung fehlen. | lack of creative ideas for healthy and varied diet |
| 38 | dass meine Ernährung einseitig und wenig abwechslungsreich geworden ist. | dissatisfaction with unbalanced and sparsely varied diet |
| 39 | meine Ernährung dauerhaft umstellen zu müssen. | permanent change of diet |
|  | |  |
| **2.3 Motivational Aspects** | |  |
| *Im Zusammenhang mit meinem Schwangerschaftsdiabetes…* | |  |
| 40 | erleichtert es mich, dass ich durch Ernährung und Bewegung die Entwicklung meines ungeborenen Kindes positiv beeinflussen kann. | positive influence on fetal development via diet and physical activity as a relief |
| 41 | erleichtert es mich, dass ich durch mehr Bewegung bessere Blutzuckerwerte erzielen kann. | better blood sugar readings through physical activity as a relief |
| 42 | nutze ich Bewegung und Sport regelmäßig, um meinen Blutzuckerspiegel zu senken. | utilization of physical activity to lower blood sugar levels |
| 43 | ist die Gesundheit meines ungeborenen Kindes ein großer Ansporn für mich, meine alltäglichen Gewohnheiten umzustellen. | fetal health as an incentive to change everyday habits |
| 44 | motiviert mich diese Erkrankung dazu, mir gesunde Gewohnheiten anzueignen und diese nach der Geburt fortzusetzen. | disease as an incentive to acquire healthy habits and pursue them after delivery |
|  | |  |
| **III. Social Domain** | |  |
| **3.1 Social Aspects** | |  |
| 1. **Support** | |  |
| *Im Zusammenhang mit meinem Schwangerschaftsdiabetes…* | |  |
| 45 | hat mir die Unterstützung meines Partners oder meines Umfelds geholfen, mit dieser Krankheit besser umzugehen. | support of partner and social environment for coping with disease |
| 46 | unterstützt mich meine Familie, indem sie sich meiner neuen Ernährungsweise anpasst. | support of family through its adaption to new diet |
| 47 | fühle ich mich gut betreut und aufgehoben bei meinen behandelnden Ärzten. | feeling of being good cared for by attending physicians |
| 48 | beruhigen und bestärken mich die Gespräche und Untersuchungen während meiner Vorsorgetermine. | reassurement and encouragement by regular medical check-ups |
| 49 | habe ich genug Möglichkeiten, mit dem medizinischen Personal zu reden und meine Fragen zu stellen. | having enough opportunities to talk to medical personal and ask them questions |
| 50 | haben mir die Aufklärungen und Schulungen durch das medizinische Personal geholfen. | coping via elucidations and training courses by medical personal |
| 51 | haben mir Informationen über diese Krankheit aus dem Internet und den Medien geholfen. | coping via information about disease out of internet and media |
| 52 | fühle ich mich ausreichend über die Ursachen und die möglichen Folgen dieser Krankheit aufgeklärt. | feeling sufficiently elucidated about the disease’s causes and potential consequences |
|  | |  |
| 1. **Participation** | |  |
| *Im Zusammenhang mit meinem Schwangerschaftsdiabetes…* | |  |
| 53 | schränken mich die zahlreichen Arztbesuche in meinem Alltag ein. | constraints in everyday life through appointments |
| 54 | habe ich durch diese Krankheit weniger Zeit für mich und meine Familie zur Verfügung. | less time for oneself and family through disease |
| 55 | verzichte ich auf den Besuch von Restaurants, Cafés oder Partys. | sacrifice for visits of restaurants, cafés and parties |
| 56 | verzichte ich auf Aktivitäten, die mir eigentlich Freude bereiten. | sacrifice for pleasurable activities |
|  | |  |
| **I. Physical Domain** | |  |
| **1.2 Treatment-related Aspects** | |  |
| 1. **Measurement** | |  |
| *Im Zusammenhang mit meinem Schwangerschaftsdiabetes…* | |  |
| 57 | fühle ich mich aufgrund hoher Blutzuckerwerte gestresst. | stress through high blood sugar levels |
| 58 | fällt mir die häufige Messung des Blutzuckerspiegels schwer. | difficulties with frequent blood sugar measurements |
| 59 | fällt es mir schwer, die Blutzuckermessungen unterwegs durchzuführen. | difficulties with blood sugar measurements on the way |
| 60 | fällt mir der technische Umgang mit dem Blutzuckermessgerät schwer. | difficulties in technical handling of blood glucose meter |
| 61 | bereitet es mir Probleme, die Blutzuckermessungen mit meiner Arbeit oder meinem Alltag zu vereinbaren. | difficulties to combine blood sugar measurements with work or everyday life |
|  | |  |
| 1. **Medication** | |  |
| *Im Zusammenhang mit meinem Schwangerschaftsdiabetes…* | |  |
| 62 | kostet es mich Überwindung und Kraft, mir Insulin zu spritzen. | insulin injection requiring effort and strength |
| 63 | fällt mir der technische Umgang mit den Insulinspritzen schwer. | difficulties in technical handling of insulin syringes |
| 64 | fällt es mir schwer, die Spritzzeiten für das Insulin einzuhalten. | difficulties in adhering with times of insulin injection |
| 65 | fällt es mir schwer, das Insulinspritzen unterwegs durchzuführen. | difficulties with insulin injections on the way |
| 66 | bereitet es mir Probleme, das Insulinspritzen mit meiner Arbeit oder meinem Alltag zu vereinbaren. | difficulties to combine insulin injections with work or everyday life |
| 67 | belasten mich die Nebenwirkungen meiner Diabetes-Medikamente. | side effects of diabetes medication as burden |
| 68 | habe ich durch einen niedrigen Blutzuckerspiegel Symptome wie Heißhunger, Unruhe, Schweißausbrüche und Zittern erlebt. | low blood sugar readings causing symptoms such as craving, restlessness, sweating and shivering |
| 69 | habe ich die Sorge, dass meine Diabetes-Medikamente meinem ungeborenen Kind schaden könnten. | concerns that diabetes medication harms fetus |

**Cave: This item list of the GDM-QOL questionnaire is not intended to be used for the assessment of the HrQoL in women with gestational diabetes at this point of time as it has not been validated.**
